# Microwave Assisted Synthesis of Some New Thiazolopyrimidine, Thiazolodipyrimidine and Thiazolopyrimidothiazolopyrimidine Derivatives with Potential Antioxidant and Antimicrobial Activity

**DOI:** 10.3390/molecules17089652

**Published:** 2012-08-13

**Authors:** Mohamed M. Youssef, Mahmoud A. Amin

**Affiliations:** 1 Chemistry Department, Faculty of Science, Cairo University, Cairo 12613, Egypt; 2 Chemistry Department, Faculty of Science, Suez Canal University, Ismailia 41522, Egypt

**Keywords:** biginelli reaction, 2-thioxopyrimidines, thiazolopyrimidines, thiazolo-dipyrimidines, thiazolopyrimidothiazolopyrimidines, antioxidant activity, antimicrobial activity

## Abstract

Biginelli reaction of ethyl acetoacetate, thiourea and the appropriate aromatic aldehyde was used to produce ethyl 4-aryl-6-methyl-2-thioxo-1,2,3,4-tetrahydro-pyrimidine-5-carboxylates, that reacted with bromomalononitrile to give ethyl 3-amino-5-aryl-2-cyano-7-methyl-5*H*-thiazolo[3,2-*a*]pyrimidine-6-carboxylates rather than the isomeric 7*H*-thiazolo[3,2-*a*]pyrimidines. Thiazolopyrimidine derivatives reacted with carbon disulphide to yield ethyl 9-aryl-7-methyl-2,4-dithioxo-2,3,4,9-tetrahydro-1*H*-thiazolo[3,2-*a*:4,5-*d'*]dipyrimidine-8-carboxylates, that reacted with phenacyl bromide to produce ethyl 8-methyl-10-(4-methoxyphenyl)-3-substituted-5-thioxo-2(un)subatituted-10*H*-thiazolo[3'',2'':1',2']pyrimido[4',5':4,5]thiazolo[3,2-*a*]pyrimidine-9-carboxylates. The aforementioned reactions were carried out using both conventional chemical methods and with the assistance of microwave irradaition. Comparison between both methods showed that the microwave assisted method is preferable because of the time reduction and yield improvements achieved. The new compounds were tested for their biological activity as antioxidants, antibacterial or antifungal agents. Some of the new compounds were found to have moderate to good antioxidant and antimicrobial activities.

## 1. Introduction

Thiazolopyrimidenes have been of interest due to their ability to inhibit 2-methylerythritol-2,4-cyclodiphosphate synthase [1]. They have been also used as analgesic and antiparkinsonian agents [2], modulators of Transient Receptor Potential Vanilloid–receptor 1 (TRPV1) [3], anticancer agents [4,5,6], pesticides [7], phosphate inhibitors [8,9], acetylcholinesterase inhibitors [10] and antimicrobial susbtances [11,12,13].

The microwave technique has several advantages over conventional methods of synthesis. Reduced reaction times [14,15,16,17], less effects on the environment and better reaction yields are some of the common advantages of using microwave irradaition. In the present research, we used both the microwave technique as well as conventional methods to prepare some thiazolopyrimidine, thiazolodipyrimidine and thiazolopyrimidothiazolopyrimidines derivatives with expected biological activity.

## 2. Results and Discussion

### 2.1. Chemistry

Prompted by the aforesaid biological and medicinal activities, samples of differently substituted thiazolopyrimidines and thiazolodipyrimidines were synthesized, using both conventional chemical methods and microwave irradiation assistance. The reaction of the precursors, ethyl 4-aryl-6-methyl-2-thioxo-1,2,3,4-tetrahydropyrimidine-5-carboxylates **1a**–**d**, with some bifunctional reagents seems to be a facile and convenient route for the synthesis of such targets. The newly synthesized compounds were tested for their antioxidant and antimicrobial activities.

**Scheme 1:**
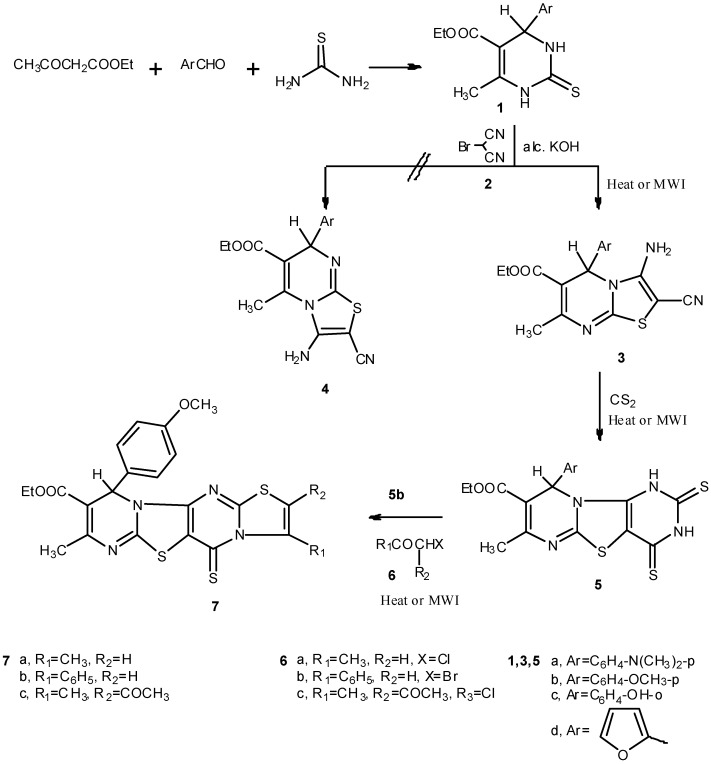
Synthesis of pyrimidinethiones, thiazolopyrimidines, thiazolodipyrimidines and thiazolopyrimidothiazol opyrimidines.

The precursor pyrimidine derivatives **1a**–**d** were prepared by the acid catalyzed condensation of ternary mixtures of aromatic aldehydes, ethyl acetoacetate and thiourea in ethanol containing a catalytic amount of hydrochloric acid, commonly known as Biginelli reaction [18,19,20] (Scheme 1). Compounds **1a**–**d**, prepared by Biginelli's method, showed correct values of elemental analyses, as well as compatible spectroscopic data.

Treating each of **1a**–**d** with bromomalononitrile (**2**) in an ethanol solution containing potassium hydroxide yielded in each case a single product which could in principle be formulated to be either the 5*H*-thiazolo[3,2-*a*]pyrimidine structure **3** or the isomeric 7*H*-thiazolo[3,2-*a*]pyrimidine structure **4** (Scheme 1). Our preference for structure **3** over structure **4** was firstly based on comparison of the ^1^H-NMR spectral data for compounds **1** and **3**. Thus, the ^1^H-NMR spectrum of **3b** showed, in addition to the ethyl ester, methoxy, aromatic and NH_2_ proton signals, a singlet (3H) at δ 2.30 ppm assigned to the CH_3_ protons and a singlet (1H) at δ 6.31 assigned to the pyrimidine H-5. The appearance of the CH_3_ proton signal of **3b** in the same position as that for the CH_3_ proton signal in **1b**, and also the downfield shift for the pyrimidine H-5 in **3b** compared with the pyrimidine H-4 in **1b**, which appeared at δ = 5.12 ppm, indicates that the moiety around H-5 in **3b** differs from that around H-4 in **1b**. Also, the moiety around CH_3_ at C-7 in **3b** is almost similar to that around CH_3_ at C-6 in **1b**. Consequently, structure **3** could be tentatively assigned for the reaction products. Structure **4** would be expected to show different δ values for the CH_3_ groups in **4b** and **1b**, and similar δ values for H-7 in **4b** and H-4 in **1b**. More conclusive evidence for structure **3** was based on carrying out a NOE experiment on compound **3b**.

Structure **4**, having a CH_3_ group at C-5 and NH_2_ at C-3 in close proximity, would show a change in CH_3_ position signal due to NOE. Actually, upon performing the NOE experiment, the position of the CH_3_ group was not affected, which indicates that CH_3_ and NH_2_ are not close to each other, thus suggesting structure **3** for the reaction product. 

Compounds **3**, as typical enaminonitriles, could be used as precursors for the preparation of thiazolodipyrimidines. Thus, heating under reflux a mixture of each of **3a**–**d** with an excess of carbon disulphide yielded, in each case, the corresponding 9-aryl-2,4-dithioxo-7-methylthiazolo[3,2-a:4,5-d]dipyrimidine-8-carboxylate **5a**–**d** (Scheme 1).

Finally, compound **5b** reacted with α-halocarbonyl compounds, namely chloroacetone, phenacyl bromide and 3-chloropentane-2,4-dione (compounds **6a**–**c**), respectively, by heating in ethanolic potassium hydoxide solution to produce the respective thiazolo[3'',2'':1',2']pyrimido[4',5':4,5]thiazolo-[3,2-*a*]pyrimidine-9-carboxylate derivatives **7a**–**c** (Scheme 1). Besides giving correct elemental analyses and compatible spectral data (see the Experimental), structure **7** was assigned to the reaction products based on the behaviour of a similar structure reported in literature by Hafez *et al. *[21].

Our research group has recently [22,23,24,25] been interested in performing synthesis of some heterocyclic compounds under environmentally friendly, time saving microwave-assisted conditions. Accordingly, we re-synthesized the previously described compounds **1a**–**d**, **3a**–**d**, **5a**–**d**, and **7a**–**c** under microwave conditions, aiming to increase reaction yields and reduce the reaction times. The results of these preparations indicated that reaction yields were increased by 17–23% compared to the conventional conditions. Reaction times were also significantly reduced. Table 1 summarizes the benefits of using microwave conditions for the synthesis of the above-mentioned compounds.

**Table 1:** Comparison between traditional methods and microwave assisted methods of synthesis of compounds **1a**–**d**, **3a**–**d**, **5a**–**d**, and **7a**–**c**.

Compound no.	Reaction Yield %		Reaction Time	
	Microwave	Conventional Method	Microwave	Conventional Method
**1a**	82	55	5 min	3 h
**1b**	87	58	5 min	3 h
**1c**	70	42	5 min	3 h
**1d**	62	35	5 min	3 h
**3a**	81	56	10 min	Overnight
**3b**	85	48	10 min	Overnight
**3c**	85	53	10 min	Overnight
**3d**	68	40	10 min	Overnight
**5a**	82	50	15 min	8 h
**5b**	80	53	15 min	8 h
**5c**	79	44	15 min	8 h
**5d**	69	37	15 min	8 h
**7a**	74	43	5 min	3 h
**7b**	88	52	5 min	3 h
**7c**	83	58	5 min	3 h

### 2.2. Biological Evaluation

#### 2.2.1. Antioxidant Screening

The newly synthesized compounds were tested for antioxidant activity as reflected in their ability to inhibit lipid peroxidation in rat brain and kidney homogenates and rat erythrocyte hemolysis. The pro-oxidant activities of the aforementioned compounds were assessed by their effects on bleomycin-induced DNA damage. Table 2 shows the antioxidant assays by erythrocyte hemolysis, which reveals that compounds **3a** and **3b** showed interesting antioxidant activity in the lipid peroxidation assays and considerable inhibitory activity in the hemolysis assay. Compounds **3c**, **3d**, **7a**, **7b** and **7c** showed moderate antioxidant and inhibitory activity. Table 3 shows the antioxidant assay by ABTS method. Compounds **3a**, **3b**, **3c** and **3d** showed potent antioxidant activity.

**Table 2:** Antioxidant assays by erythrocyte hemolysis (A/B × 100).

Compounds	Absorbance of samples (A)	Hemolysis (%)
Complete hemolysis with distilled water (B)	0.660	-
Ascorbic acid	0.026	3.93
**1a**	0.082	10.33
**1b**	0.075	9.12
**1c**	0.090	13.01
**1d**	0.092	14.12
**3a**	0.035	5.22
**3b**	0.031	4.68
**3c**	0.045	6.92
**3d**	0.051	8.02
**5a**	0.115	21.60
**5b**	0.112	19.25
**5c**	0.132	24.07
**5d**	0.130	23.12
**7a**	0.042	6.36
**7b**	0.045	6.81
**7c**	0.043	6.51

**Table 3:** Antioxidant assays by ABTS method [Abs. (control) − Abs.(test)/Abs.(control) × 100].

Compounds	Absorbance of sample	Inhibition (%)
**ABTS control**	0.54	0
**Ascorbic acid**	0.06	88.8
**1a**	0.20	63.0
**1b**	0.23	57.4
**1c**	0.29	46.3
**1d**	0.28	48.1
**3a**	0.10	81.5
**3b**	0.12	77.7
**3c**	0.15	72.2
**3d**	0.13	75.9
**5a**	0.45	16.6
**5b**	0.42	22.2
**5c**	0.48	11.1
**5d**	0.43	20.3
**7a**	0.17	68.5
**7b**	0.19	64.8
**7c**	0.16	70.3

All compounds have been tested on bleomycin-dependent DNA damage. The results, shown in Table 4, indicate that compounds **3a**–**d** may have some protective activity towards DNA from the damage induced by bleomycin.

**Table 4:** Assays for bleomycin-dependent DNA damage.

Compound	Absorbance of Samples
**Ascorbic acid**	0.020
**3a**	0.026
**3b**	0.029
**3c**	0.037
**3d**	0.033

#### 2.2.2. Antimicrobial Evaluation

The newly synthesized heterocyclic compounds listed in Table 4 were tested for their antimicrobial activity against the following microorganisms: *Escherichia coli*, *Pseudomonas putida*, *Bacillus subtilis*, *Streptococcus lactis*, *Aspergillus niger*, *Penicillium *sp. and *Candida albicans*. The preliminary screening of the investigated compounds was performed using the filter paper disc-diffusion method. The most active compounds were **1a**, **1b**, **3a**, **3b**, **5a**, and **5b**, which showed moderate to slight inhibitory action towards the microorganisms. The rest of compounds showed slight to no sensitivity at all to the tested organisms, and the results are summarized in Table 5.

**Table 5:** Antimicrobial activities of the newly synthesized compounds.

Compd. No.	Inhibition zone (mm)						
	Gram-negative		Gram-positive		Fungi Yeast		
	*E. coli*	*P. putida*	*B. subtilis*	*S. lactis*	*A. niger*	*P. *sp*.*	*C. albicans*
**1a**	12	8	6	8	5	5	0
**1b**	14	9	6	7	4	2	0
**1c**	6	3	0	0	2	2	0
**1d**	3	2	0	0	0	0	0
**3a**	15	11	9	6	7	5	0
**3b**	12	7	7	5	7	5	0
**3c**	0	0	0	0	0	0	0
**3d**	2	2	0	0	0	0	0
**5a**	10	7	8	6	4	3	
**5b**	11	8	9	6	5	2	
**5c**	4	2	0	0	0	0	0
**5d**	3	0	0	0	0	0	0
**7a**	0	0	0	0	0	0	0
**7b**	0	0	0	0	0	0	0
**7c**	0	0	0	0	0	0	0
Chloram-phenicol^®^	22	21	18	19	20	12	0
Ampicillin^®^	24	20	19	22	24	14	14

*E. coli* = *Escherichia coli*; *P. putida* = *Pseudomonas putida*; *B. subtilis* = *Bacillus subtilis*; *S. lactis* = *Streptococcus lactis*; *A. niger* = *Aspergillus niger*; *P. *sp. = *Penicillium *sp.; *C. albicans* = *Candida albicans*; The sensitivity of microorganisms to the tested compounds is identified in the following manner *: Highly sensitive = Inhibition zone: 15–20 mm; Moderately sensitive = Inhibition zone: 10–15 mm; Slightly sensitive = Inhibition zone: 1–10 mm; Not sensitive = Inhibition zone: 0 mm; * each result represents the average of triplicate readings.

## 3. Experimental

### 3.1. General

Melting points were determined in open glass capillaries on a Gallenkamp melting point apparatus and are uncorrected. IR spectra (KBr discs) were recorded on a Shimadzu FTIR-8201PC Spectrophotometer. ^1^H-NMR and ^13^C-NMR spectra were recorded on a Varian Mercury 300 MHz and a Varian Gemini 200 MHz spectrometers using TMS as an internal standard and DMSO-*d_6_*, and as a solvent. Chemical shifts were expressed as d (ppm) units. Mass spectra were recorded on a Shimadzu GCMS-QP1000EX instrument using an inlet type sample injection at 70 eV. The Microanalytical Center of Cairo University performed the microanalyses. Microwave reactions were performed with a Millstone Organic Synthesis Unit (MicroSYNTH with touch control terminal) with a continuous focused microwave power delivery system in a pressure glass vessel (10 mL) sealed with a septum under magnetic stirring. The temperature of the reaction mixture was monitored using a calibrated infrared temperature control under the reaction vessel, and control of the pressure was performed with a pressure sensor connected to the septum of the vessel.

#### 3.1.1. Ethyl 4-aryl-6-methyl-2-thioxo-1,2,3,4-tetrahydropyrimidine-5-carboxylates **1a**–**d**

*Method A:* A solution of thiourea (0.76 g, 0.01 mol), ethyl acetoacetate (1.30 g, 0.01 mol) and the appropriate aromatic aldehyde (0.01 mol) in ethanol (50 mL) in the presence of conc. HCl (5 mL) was heated under reflux for 3 h. The reaction mixture was then allowed to stand at room temperature overnight whereby the solid precipitate so-formed was collected by filtration, washed with ethanol and crystallized from ethanol.

*Method B:* The same reactants of *Method A* were heated in microwave oven at 140 °C for 5 min. The reaction mixture was treated in a similar manner to *Method A* to obtain compounds **1a**–**d**.

*Ethyl 4-(4-(dimethylamino)phenyl)-6-methyl-2-thioxo-1,2,3,4-tetrahydropyrimidine-5-carboxylate *(**1a**) was obtained as pale green crystals, m.p. 201 °C. ^1^H-NMR: d (ppm) 1.10 (t, 3H, CH_3_, *J =* 4 Hz), 2.26 (s, 3H, CH_3_), 2.87 (s, 6H, N(CH_3_)_2_), 3.97 (q, 2H, CH_2_, *J =* 4 Hz), 5.02 (s, 1H, pyrimidine H-4), 6.65 (d, 2H, Ar-H, *J =* 5 Hz), 7.00 (d, 2H, Ar-H, *J =* 5 Hz), 9.55 (s, 1H, NH, D_2_O exchangeable) and 10.23 (s, 1H, NH, D_2_O exchangeable). ^13^C-NMR: d (ppm) 14.1 (CH_3_), 17.2 (CH_3_), 53.5 (pyrimidine C-4), 59.5 (N(CH_3_)_2_), 67.3 (CH_2_), 101.2, 112.2, 127.1, 131.2, 144.4, 150.0 (aromatic carbons + pyrimidine C-5 and C-6), 165.3 (C=S) and 173.8 (C=O). IR (KBr) n: 3268, 3188 (NH), 1718 (C=O), 1605, 1500 cm^−1^ (Aromatic C=C). MS (70 eV): (M^+^) *m/z* 319 (11.2%). Anal. Calcd. for C_16_H_20_N_3_O_2_S (319.42): C(60.16%), H(6.63%), N(13.16%), S(10.04); Found: C(60.3%), H(6.8%), N(13.4%), S(10.0%).

*Ethyl 4-(4-methoxyphenyl)-6-methyl-2-thioxo-1,2,3,4-tetrahydropyrimidine-5-carboxylate *(**1b**) was obtained as yellow crystals, m.p. 144 °C. ^1^H-NMR: d (ppm) 1.22 (t, 3H, CH_3_, *J =* 4 Hz), 2.26 (s, 3H, CH_3_), 3.25 (s, 3H, OCH_3_), 3.95 (q, 2H, CH_2_, *J =* 4 Hz), 5.12 (s, 1H, pyrimidine H-4), 6.81 (d, 2H, Ar-H, *J =* 6 Hz), 7.11 (d, 2H, Ar-H, *J =* 6 Hz), 9.50 (s, 1H, NH, D_2_O exchangeable) and 10.18 (s, 1H, NH, D_2_O exchangeable). ^13^C-NMR: d (ppm) 14.0 (CH_3_), 17.4 (CH_3_), 53.5 (pyrimidine C-4), 63.9 (OCH_3_), 68.8 (CH_2_), 103.4, 113.1, 129.7, 134.2, 145.9 153.2 (aromatic carbons + pyrimidine C-5 and C-6), 165.6 (C=S) and 174.0 (C=O). IR (KBr) n: 3272, 3185 (NH), 1718 (C=O), 1603, 1506 cm^−1^ (Aromatic C=C). MS (70 eV): (M^+^) *m/z* 306 (8.5%). Anal. Calcd. for C_15_H_18_N_2_O_3_S (306.38): C(58.80%), H(5.92%), N(9.14%), S(10.47); Found: C(59.0%), H(5.9%), N(9.3%), S(10.7%).

*Ethyl 4-(2-hydroxyphenyl)-6-methyl-2-thioxo-1,2,3,4-tetrahydropyrimidine-5-carboxylate *(**1c**) was obtained as beige crystals, m.p. 235 °C. ^1^H-NMR: d (ppm) 1.18 (t, 3H, CH_3_, *J =* 4 Hz), 2.19 (s, 3H, CH_3_), 3.95 (q, 2H, CH_2_, *J =* 4 Hz), 5.10 (s, 1H, pyrimidine H-4), 6.73–7.07 (m, 4H, Ar-H), 8.40 (s, 1H, OH, D_2_O exchangeable), 9.55 (s, 1H, NH, D_2_O exchangeable) and 10.12 (s, 1H, NH, D_2_O exchangeable). ^13^C-NMR: d (ppm) 13.5 (CH_3_), 16.8 (CH_3_), 53.5 (pyrimidine C-4), 67.6 (CH_2_), 102.7, 112.6, 115.3, 122.0 127.7, 131.9, 144.6 150.9 (aromatic carbons + pyrimidine C-5 and C-6), 165.2 (C=S) and 172.6 (C=O). IR (KBr) n: 3280–3080 (broad, OH+NH), 1722(C=O), 1600, 1501 cm^−1^ (Aromatic C=C). MS (70 eV): (M^+^) *m/z* 292 (14.1%). Anal. Calcd. for C_14_H_16_N_2_O_3_S (292.09): C(57.52%), H(5.52%), N(9.58%), S(10.97); Found: C(57.7%), H(5.8%), N(9.5%), S(11.1%).

*Ethyl 4-(furan-2-yl)-6-methyl-2-thioxo-1,2,3,4-tetrahydropyrimidine-5-carboxylate *(**1d**) was obtained as brown crystals, m.p. 176 °C. ^1^H-NMR: d (ppm) 1.25 (t, 3H, CH_3_, *J =* 4 Hz), 2.22 (s, 3H, CH_3_), 4.20 (q, 2H, CH_2_, *J =* 4 Hz), 5.23 (s, 1H, pyrimidine H-4), 6.44 (d, 1H, furan-H), 6.56 (m, 1H, furan-H), 7.41 (d, 1H, furan-H), 9.34 (s, 1H, NH, D_2_O exchangeable) and 10.20 (s, 1H, NH, D_2_O exchangeable). ^13^C-NMR: d (ppm) 14.3 (CH_3_), 17.5 (CH_3_), 57.5 (pyrimidine C-4), 67.2 (CH_2_), 106.2, 110.9, 143.1, 145.3, 151.0 152.9 (furan carbons + pyrimidine C-5 and C-6), 165.8 (C=S) and 173.0 (C=O). IR (KBr) n: 3275, 3183 (NH), 1720 (C=O), 1607, 1500 cm^−1^ (aromatic C=C). MS (70 eV): (M^+^) *m/z* 266 (6.3%). Anal. Calcd. for C_12_H_14_N_2_O_3_S (266.32): C(54.12%), H(5.30%), N(10.52%), S(12.04); Found: C(54.1%), H(5.4%), N(10.4%), S(12.0%).

#### 3.1.2. Ethyl 3-amino-5-aryl-2-cyano-7-methyl-5*H*-thiazolo[3,2-a]pyrimidine-6-carboxylates (**3a**–**d**)

*Method A: *To a warm ethanolic potassium hydroxide solution [prepared by dissolving KOH (0.56 g, 0.01 mol) in ethanol (50 mL)] of each of **1a**–**d** (0.01 mol), bromomalononitrile **2** (1.45 g, 0.01 mol) was added portion-wise with stirring. The reaction mixture was then left overnight at room temperature, whereby the solid product that separated upon dilution with water was filtered off and crystallized from the proper solvent.

*Method B: *The same reactants of method A were heated at 140 °C in microwave oven for 10 min. The reaction mixture was treated in a similar manner to method A to obtain compounds **3a**–**d**.

*Ethyl 3-amino-2-cyano-5-(4-(dimethylamino)phenyl)-7-methyl-5H-thiazolo[3,2-a]pyrimidine-6-carboxylate* (**3a**) was crystallized from dil. dioxane as yellowish green crystals, m.p. 218 °C. ^1^H-NMR: d (ppm) 1.35 (t, 3H, CH_3_, *J =* 4 Hz), 2.34 (s, 3H, CH_3_), 2.95 (s, 6H, N(CH_3_)_2_), 4.15 (q, 2H, CH_2_, *J =* 4 Hz), 6.22 (s, 1H, pyrimidine H-5), 6.60 (d, 2H, Ar-H, *J =* 5 Hz), 6.97 (d, 2H, Ar-H, *J =* 5 Hz) and 8.78 (s, 2H, NH_2_, D_2_O exchangeable). ^13^C-NMR: d (ppm) 14.5 (CH_3_), 17.3 (CH_3_), 58.8 (pyrimidine C-5), 59.7 (N(CH_3_)_2_), 68.4 (CH_2_), 107.3 (CN), 112.2, 117.3, 127.1, 132.2, 149.4, 154.6, 157.3, 158.1, 158.9 (aromatic carbons + pyrimidine C-6 and C-7, C-8a + thiazole C-2, C-3), and 171.6 (C=O). IR (KBr) n: 3310, 3244 (NH_2_), 2217 (CN), 1724 (C=O), 1605, 1500 cm^−1^ (aromatic C=C). MS (70 eV): (M^+^) *m/z* 383 (7.3%). Anal. Calcd. for C_19_H_21_N_5_O_2_S (383.14): C(59.51%), H(5.52%), N(18.26%), S(8.36); Found: C(59.6%), H(5.8%), N(18.3%), S(8.3%).

*Ethyl 3-amino-2-cyano-5-(4-methoxyphenyl)-7-methyl-5H-thiazolo[3,2-a]pyrimidine-6-carboxylate* (**3b**) was crystallized from ethanol as yellow crystals, m.p. 220 °C. ^1^H-NMR: d (ppm) 1.30 (t, 3H, CH_3_, *J =* 4 Hz), 2.30 (s, 3H, CH_3_), 3.85 (s, 3H, OCH_3_), 4.18 (q, 2H, CH_2_, *J =* 4 Hz), 6.31 (s, 1H, pyrimidine H-5), 6.80 (d, 2H, Ar-H, *J =* 6 Hz), 7.15 (d, 2H, Ar-H, *J =* 6 Hz), and 8.40 (s, 2H, NH_2_, D_2_O exchangeable). ^13^C-NMR: d (ppm) 14.8 (CH_3_), 17.4 (CH_3_), 59.6 (pyrimidine C-5), 62.1 (OCH_3_), 67.4 (CH_2_), 108.1 (CN), 113.1, 118.8, 129.0, 134.1, 151.4, 155.1, 157.8, 158.7, 159.3 (aromatic carbons + pyrimidine C-6 and C-7, C-8a + thiazole C-2, C-3), and 171.0 (C=O). IR (KBr) n: 3300, 3230 (NH_2_), 2210 (CN), 1720 (C=O), 1605, 1500 cm^−1^ (aromatic C=C). MS (70 eV): (M^+^) *m/z* 370 (8.1%). Anal. Calcd. for C_18_H_18_N_4_O_3_S (370.43): C(58.36%), H(4.90%), N(15.12%), S(8.66); Found: C(58.5%), H(4.8%), N(15.3%), S(8.5%).

*Ethyl 3-amino-2-cyano-5-(2-hydroxyphenyl)-7-methyl-5H-thiazolo[3,2-a]pyrimidine-6-carboxylate *(**3c**) was crystallized from ethanol as yellow crystals, m.p. 277 °C. ^1^H-NMR: d (ppm) 1.32 (t, 3H, CH_3_, *J =* 4 Hz), 2.34 (s, 3H, CH_3_), 4.05 (q, 2H, CH_2_, *J =* 4 Hz), 6.24 (s, 1H, pyrimidine H-5), 6.80–7.18 (m, 4H, Ar-H), 8.15 (s, 1H, OH, D_2_O exchangeable) and 8.53 (s, 2H, NH_2_, D_2_O exchangeable). ^13^C-NMR: d (ppm) 14.3 (CH_3_), 17.1 (CH_3_), 58.8 (pyrimidine C-5), 67.1 (CH_2_), 107.3 (CN), 112.3, 116.1, 122.1, 126.3, 131.3, 137.1, 152.1, 157.1, 157.9, 158.8, 159.5 (aromatic carbons + pyrimidine C-6 and C-7, C-8a + thiazole C-2, C-3), and 173.0 (C=O). IR (KBr) n: 3320–3118 (broad, OH+NH_2_), 2210 (CN), 1716 (C=O), 1600, 1500 cm^−1^ (aromatic C=C). MS (70 eV): (M^+^) *m/z* 356 (12.5%). Anal. Calcd. for C_17_H_16_N_4_O_3_S (356.40): C(57.29%), H(4.52%), N(15.72%), S(9.00); Found: C(57.5%), H(4.6%), N(15.6%), S(9.1%).

*Ethyl 3-amino-2-cyano-5-(furan-2-yl)-7-methyl-5H-thiazolo[3,2-a]pyrimidine-6-carboxylate *(**3d**) was crystallized from dioxane as brown crystals, m.p. 345 °C. ^1^H-NMR: d (ppm) 1.27 (t, 3H, CH_3_, *J =* 4 Hz), 2.21 (s, 3H, CH_3_), 4.25 (q, 2H, CH_2_, *J =* 4 Hz), 6.11 (s, 1H, pyrimidine H-5), 6.45 (d, 1H, furan-H), 6.61 (m, 1H, furan-H), 7.43 (d, 1H, furan-H) and 8.87 (s, 2H, NH_2_, D_2_O exchangeable).^ 13^C-NMR: d (ppm) 14.7 (CH_3_), 17.5 (CH_3_), 62.3 (pyrimidine C-5), 67.4 (CH_2_), 107.1 (CN), 112.6, 114.1, 127.3, 135.1, 139.6, 143.1, 145.3, 151.0 152.9 (furan carbons + pyrimidine C-6 and C-7, C-8a + thiazole C-2, C-3) and 173.3 (C=O). IR (KBr) n: 3310, 3244 (NH_2_), 2210 (CN), 1722 (C=O), 1605, 1510 cm^−1^ (aromatic C=C). MS (70 eV): (M^+^) *m/z* 330 (4.8%). Anal. Calcd. for C_15_H_14_N_4_O_3_S (330.36): C(54.53%), H(4.27%), N(16.96%), S(9.71); Found: C(54.4%), H(4.4%), N(16.7%), S(9.8%).

#### 3.1.3. Ethyl 9-aryl-7-methyl-2,4-dithioxo-2,3,4,9-tetrahydro-1*H*-thiazolo[3,2-a:4,5-d']dipyrimidine-8-carboxylates **5a**–**d**

*Method A*: Each of compounds **3a**–**d** (0.01 mol) was heated under reflux with an excess of carbon disulphide (10 mL) for 8 h. The reaction mixture was then cooled, and the solid that precipitated was filtered at the pump and crystallized from the proper solvent.

*Method B: *The same reactants of method A were heated at 140 °C in microwave oven for 15 min. The reaction mixture was treated in a similar manner to method A to obtain compounds **5a**–**d**.

*Ethyl 9-(4-(dimethylamino)phenyl)-7-methyl-2,4-dithioxo-2,3,4,9-tetrahydro-1H-thiazolo-[3,2-a:4,5-d']dipyrimidine-8-carboxylate *(**5a**) was crystallized from dilute ethanol as grey crystals, m.p. 248 °C. ^1^H-NMR: d (ppm) 1.10 (t, 3H, CH_3_, *J =* 4 Hz), 2.26 (s, 3H, CH_3_), 2.84 (s, 6H, N(CH_3_)_2_), 3.95 (q, 2H, CH_2_, *J =* 4 Hz), 5.88 (s, 1H, pyrimidine H-9), 6.60 (d, 2H, Ar-H, *J =* 5 Hz), 6.95 (d, 2H, Ar-H, *J =* 5 Hz), 11.35 (s, 1H, NH, D_2_O exchangeable) and 12.12 (s, 1H, NH, D_2_O exchangeable). ^13^C-NMR: d (ppm) 14.2 (CH_3_), 17.8 (CH_3_), 53.5 (pyrimidine C-9), 59.6 (N(CH_3_)_2_), 67.3 (CH_2_), 109.2, 111.3, 127.1, 132.2, 148.2, 154.3, 156.2 157.1, 161.9 (aromatic carbons + pyrimidine C-5a, C-7, C-8 + thiazole C-4a, C-10a), 171.3 (C=S), 175.1 (C=O) and 181.4 (C=S). IR (KBr) n: 3305, 3200 (NH_2_), 1712 (C=O), 1605, 1500 cm^−1^ (aromatic C=C). MS (70 eV): (M^+^) *m/z* 459 (3.2%). Anal. Calcd. for C_20_H_21_N_5_O_2_S_3_ (459.61): C(52.26%), H(4.61%), N(15.24%), S(20.93); Found: C(52.3%), H(4.7%), N(15.5%), S(20.8%).

*Ethyl 9-(4-methoxyphenyl)-7-methyl-2,4-dithioxo-2,3,4,9-tetrahydro-1H-thiazolo[3,2-a:4,5-d']-dipyrimidine-8-carboxylate *(**5b**) was crystallized from ethanol as beige crystals, m.p. 243 °C.^1^H-NMR: d (ppm) 1.13 (t, 3H, CH_3_, *J =* 4 Hz), 2.32 (s, 3H, CH_3_), 3.22 (s, 3H, OCH_3_), 4.10 (q, 2H, CH_2_, *J =* 4 Hz), 5.95 (s, 1H, pyrimidine H-9), 6.80 (d, 2H, Ar-H, *J =* 6 Hz), 7.12 (d, 2H, Ar-H, *J =* 6 Hz), 11.30 (s, 1H, NH, D_2_O exchangeable) and 12.10 (s, 1H, NH, D_2_O exchangeable). ^13^C-NMR: d (ppm) 14.1 (CH_3_), 18.6 (CH_3_), 61.0 (pyrimidine C-9), 63.2 (OCH_3_), 67.3 (CH_2_), 110.4, 114.6, 130.0, 133.8 149.8, 155.2, 156.4, 157.9, 162.2 (aromatic carbons + pyrimidine C-5a, C-7, C-8 + thiazole C-4a, C-10a), 171.1 (C=S), 175.0 (C=O) and 181.5 (C=S). IR (KBr) n: 3310, 3200 (NH), 1718 (C=O), 1605, 1500 cm^−1^ (aromatic C=C). MS (70 eV): (M^+^) *m/z* 446 (4.0%). Anal. Calcd. for C_19_H_18_N_4_O_3_S_3_ (446.57): C(51.10%), H(4.06%), N(12.55%), S(21.54); Found: C(51.0%), H(4.2%), N(12.5%), S(21.8%).

*Ethyl 9-(2-hydroxyphenyl)-7-methyl-2,4-dithioxo-2,3,4,9-tetrahydro-1H-thiazolo[3,2-a:4,5-d']-dipyrimidine-8-carboxylate *(**5c**) was crystallized from dioxane as pale green crystals, m.p. 295 °C. ^1^H-NMR: d (ppm) 1.28 (t, 3H, CH_3_, *J =* 4 Hz), 2.25 (s, 3H, CH_3_), 4.16 (q, 2H, CH_2_, *J =* 4 Hz), 6.11 (s, 1H, pyrimidine H-9), 6.80–7.20 (m, 4H, Ar-H), 8.22 (s, 1H, OH, D_2_O exchangeable), 11.40 (s, 1H, NH, D_2_O exchangeable) and 12.25 (s, 1H, NH, D_2_O exchangeable). ^13^C-NMR: d (ppm) 14.3 (CH_3_), 17.1 (CH_3_), 58.8 (pyrimidine C-5), 67.1 (CH_2_), 107.3 (CN), 107.9, 112.4, 120.1, 127.4, 133.3, 138.1, 154.0, 158.1, 158.9, 159.4, 161.0 (aromatic carbons + pyrimidine C-5a, C-7, C-8 + thiazole C-4a, C-10a), 171.4 (C=S), 174.8 (C=O) and 180.7 (C=S). IR (KBr) n: 3300, 3230 (NH_2_), 1710 (C=O), 1600, 1500 cm^−1^ (aromatic C=C). MS (70 eV): (M^+^) *m/z* 432 (5.0%). Anal. Calcd. for C_18_H_16_N_4_O_3_S_3_ (432.54): C(49.98%), H(3.73%), N(12.95%), S(22.24); Found: C(49.8%), H(3.8%), N(13.1%), (22.1%).

*Ethyl 9-(furan-2-yl)-7-methyl-2,4-dithioxo-2,3,4,9-tetrahydro-1H-thiazolo[3,2-a:4,5-d']dipyrimidine-8-carboxylate *(**5d**) was crystallized from dioxane as dark green crystals, m.p. 255 °C. ^1^H-NMR: d (ppm) 1.25 (t, 3H, CH_3_, *J =* 4 Hz), 2.15 (s, 3H, CH_3_), 4.00 (q, 2H, CH_2_, *J =* 4 Hz), 6.11 (s, 1H, pyrimidine H-9), 6.51 (d, 1H, furan-H), 6.78 (m, 1H, furan-H), 7.55 (d, 1H, furan-H), 11.40 (s, 1H, NH, D_2_O exchangeable) and 12.25 (s, 1H, NH, D_2_O exchangeable).^ 13^C-NMR: d (ppm) 14.5 (CH_3_), 17.1 (CH_3_), 62.1 (pyrimidine C-9), 66.8 (CH_2_), 106.6, 110.0, 123.3, 134.8, 138.7, 142.7, 144.9, 151.4 157.9 (furan carbons + pyrimidine C-5a, C-7, C-8 + thiazole C-4a, C-10a) 171.8 (C=S), 173.9 (C=O) and 181.2 (C=S). IR (KBr) n: 3310, 3244 (2NH), 2210 (CN), 1722 cm^−1^ (C=O), 1605, 1510 (aromatic C=C). MS (70 eV): (M^+^) *m/z* 406 (4.3 %). Anal. Calcd. for C_16_H_14_N_4_O_3_S_3_ (406.50): C(47.27%), H(3.47%), N(13.78%), S(23.66); Found: C(47.0%), H(3.4%), N(13.8%), S(23.8%).

#### 3.1.4. Ethyl 8-methyl-10-(4-methoxyphenyl)-3-substituted-5-thioxo-2-(un)substituted-10*H*-thiazolo-[3'',2'':1',2']pyrimido[4',5':4,5]thiazolo[3,2-a]pyrimidine-9-carboxylates **7a**–**c**

*Method A: *A solution of **5b** (4.46 g, 0.01 mol) with each of chloroacetone (0.92 g, 0.1 mol), phenacyl bromide (1.99 g, 0.01 mol) or 3-chloropentane-2,4-dione (1.34 g, 0.01 mol) in ethanolic potassium hydroxide solution [prepared by dissolving KOH (0.56 g, 0.01 mol) in ethanol (50 mL)] was heated under reflux for 3 h. A precipitate started to form on hot after 1 h. After cooling, the produced precipitate was filtered off, dried and crystallized from dimethyl formamide.

*Method B: *The same reactants of method A were heated at 140 °C in microwave oven for 5 min. The reaction mixture was treated in a similar manner to method A to obtain compounds **7a**–**c**.

*Ethyl 3,8-dimethyl-10-(4-methoxyphenyl)-5-thioxo-10H-thiazolo[3'',2'':1',2'] pyrimido[4',5':4,5]-thiazolo[3,2-a]pyrimidine-9-carboxylate *(**7a**) was obtained as yellow crystals, m.p. 314 °C.^1^H-NMR: d (ppm) 1.34 (t, 3H, CH_3_, *J =* 4 Hz), 2.45 (s, 3H, CH_3_), 3.11 (s, 3H, CH_3_), 3.91 (s, 3H, OCH_3_), 4.53 (q, 2H, CH_2_, *J =* 4 Hz), 5.77 (s, 1H, pyrimidine H-10), 6.94 (s, 1H, thiazole H-2), 7.15 (d, 2H, Ar-H, *J =* 6 Hz), 7.68 (d, 2H, Ar-H, *J =* 6 Hz). ^13^C-NMR: d (ppm) 14.7 (CH_3_), 19.6 (CH_3_), 23.3 (CH_3_), 58.2 (OCH_3_), 63.1 (CH_2_), 67.5 (pyrimidine C-10), 101.6, 106.4, 112.3, 124.1, 128.0, 135.8 151.3, 155.0, 157.4, 158.3, 158.5, 160.1 (aromatic carbons + pyrimidine C-6a, C-7a, C-8, C-9, C-11a, C-12a + thiazole C-2, C-3), 171.0 (C=O) and 182.5 (C=S). IR (KBr) n: 1715 (C=O), 1600, 1508 cm^−1^ (Aromatic C=C). MS (70 eV): (M^+^) *m/z* 484 (3.6%). Anal. Calcd. for C_22_H_20_N_4_O_3_S_3_ (484.57): C(54.52%), H(4.16%), N(11.56%), S(19.85); Found: C(54.34%), H(4.2%), N(11.8%), S(19.8%).

*Ethyl 8-methyl-10-(4-methoxyphenyl)-3-phenyl-5-thioxo-10H-thiazolo[3'',2'':1',2']pyrimido-[4',5':4,5]-thiazolo[3,2-a]pyrimidine-9-carboxylate *(**7b**) was obtained as orange crystals, m.p. 335 °C. ^1^H-NMR: d (ppm) 1.41 (t, 3H, CH_3_, *J =* 4 Hz), 3.17 (s, 3H, CH_3_), 3.95 (s, 3H, OCH_3_), 4.50 (q, 2H, CH_2_, *J =* 4 Hz), 5.75 (s, 1H, pyrimidine H-10), 7.04 (s, 1H, thiazole H-2), 7.22 (d, 2H, Ar-H, *J =* 6 Hz), 7.41 (d, 2H, Ar-H, *J =* 6 Hz), 7.52–7.81 (m, 5H, Ar-H). ^13^C-NMR: d (ppm) 14.6 (CH_3_), 24.7 (CH_3_), 58.0 (OCH_3_), 63.4 (CH_2_), 67.2 (pyrimidine C-10), 103.2, 106.9, 112.8, 115.6, 124.9, 126.2, 128.0, 128.9, 129.3, 135.8, 152.2, 155.1, 157.2, 158.0, 160.4 (aromatic carbons + pyrimidine C-6a, C-7a, C-8, C-9, C-11a, C-12a + thiazole C-2, C-3), 170.7 (C=O) and 183.0 (C=S). IR (KBr) n: 1718 (C=O), 1600, 1504 cm^−1^ (aromatic C=C). MS (70 eV): (M^+^) *m/z* 546 (2.3%). Anal. Calcd. for C_27_H_22_N_4_O_3_S_3_ (546.68): C(59.32%), H(4.06%), N(10.25%), S(17.60); Found: C(59.2%), H(4.2%), N(10.6%), S(17.8%).

*Ethyl 2-acetyl-3,8-dimethyl-10-(4-methoxyphenyl)-5-thioxo-10H-thiazolo[3'',2'':1',2']pyrimido-[4',5':4,5]thiazolo[3,2-a]pyrimidine-9-carboxylate *(**7c**) was obtained as yellow crystals, m.p. 308 °C. ^1^H-NMR: d (ppm) 1.35 (t, 3H, CH_3_, *J =* 4 Hz), 2.47 (s, 3H, CH_3_), 2.51 (s, 3H, CH_3_), 3.10 (s,3H,CH_3_), 3.95 (s, 3H, OCH_3_), 4.50 (q, 2H, CH_2_, *J =* 4 Hz), 5.68 (s, 1H, pyrimidine H-10), 7.00 (d, 2H, Ar-H, *J =* 6 Hz), 7.55 (d, 2H, Ar-H, *J =* 6 Hz). ^13^C-NMR: d (ppm) 12.3 (CH_3_), 14.5 (CH_3_), 20.8 (CH_3_), 25.3 (CH_3_), 58.0 (OCH_3_), 63.1 (CH_2_), 65.5 (pyrimidine C-10), 105.6, 106.2, 112.6, 124.3, 127.2, 135.6 151.0, 154.3 155.6, 157.2, 158.1, 160.4 (aromatic carbons + pyrimidine C-6a, C-7a, C-8, C-9, C-11a, C-12a + thiazole C-2, C-3), 170.4 (C=O) ), 175.3 (C=O) and 182.5 (C=S). IR (KBr) n: 1715, 1695 (2 C=O), 1600, 1508 cm^−1^ (aromatic C=C). MS (70 eV): (M^+^) *m/z* 526 (5.5%). Anal. Calcd. for C_24_H_22_N_4_O_4_S_3_ (526.08): C(54.73%), H(4.21%), N(10.64%), S(18.27); Found: C(54.5%), H(4.3%), N(10.7%), S(18.1%).

### 3.2. Antioxidant Screening

#### 3.2.1. Assay for Erythrocyte Hemolysis

Blood was obtained from rats by cardiac puncture and collected in heparinized tubes. Erythrocytes were separated from plasma and the buffy coat and washed three times with 10 volumes of 0.15 M NaCl. During the last washing, the erythrocytes were centrifuged at 2,500 rpm for 10 min to obtain a constantly packed cell preparation. Erythrocyte hemolysis was mediated by peroxyl radicals in this assay system [26]. A 10% suspension of erythrocytes in pH 7.4 phosphate-buffered saline (PBS) was added to the same volume of 200 mM 2,2′-azobis(2-amidinopropane)dihydrochloride (AAPH) solution (in PBS) containing samples to be tested at different concentrations. The reaction mixture was shaken gently while being incubated at 37 °C for 24 h. The reaction mixture was then removed, diluted with eight volumes of PBS and centrifuged at 2,500 rpm for 10 min. The absorbance A of the supernatant was read at 540 nm. Similarly, the reaction mixture was treated with eight volumes of distilled water to achieve complete hemolysis, and the absorbance B of the supernatant obtained after centrifugation was measured at 540 nm. The percentage hemolysis was calculated by the equation (1 − A/B) × 100%. The data were expressed as mean standard deviation. L-Ascorbic was used as a positive control.

#### 3.2.2. Antioxidant Activity Screening Assay—ABTS Method

For each of the investigated compounds 2 mL of 2,2'-azino-bis(3-ethylbenzthiazoline-6-sulphonic acid) (ABTS) solution (60 mM) was added to 3 M MnO_2_ solution (25 mg/mL) all prepared in phosphate buffer (pH 7, 0.1 M). The mixture was shaken, centrifuged, filtered, and the absorbance (Acontrol) of the resulting green-blue solution (ABTS radical solution) was adjusted at ca. 0.5 at l 734 nm. Then, 50 mL of 2 mM solution of the test compound in spectroscopic grade MeOH/phosphate buffer (1:1) was added. The absorbance (Atest) was measured and the reduction in color intensity was expressed as % inhibition. The % inhibition for each compound is calculated from the following equation [27]:


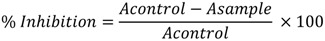


Ascorbic acid (vitamin C) was used as standard antioxidant (positive control). Blank sample was run without ABTS and using MeOH/phosphate buffer (1:1) instead of sample. Negative control sample was run with MeOH/phosphate buffer (1:1) instead of tested compound.

#### 3.2.3. Bleomycin-Dependent DNA Damage

The assay was done according to Aeschlach *et al. *[28] with minor modifications. The reaction mixture (0.5 mL) contained DNA (0.5 mg/ mL), bleomycin sulfate (0.05 mg/mL), MgCl_2_ (5 mM), FeCl_3_ (50 mM) and samples to be tested at different concentrations. L-Ascorbic acid was used as a positive control. The mixture was incubated at 37 °C for 1 h. The reaction was terminated by addition of 0.05 mL EDTA (0.1 M). The color was developed by adding 0.5 mL thiobarbituric acid (TBA) (1%, w/v) and 0.5 mL HCl (25%, v/v) followed by heating at 80 °C for 10 min. After centrifugation, the extent of DNA damage was measured by increase in absorbance at 532 nm.

### 3.3. Antimicrobial Screening

The newly synthesized heterocyclic compounds were tested for their antimicrobial activity against the following microorganisms: (a) Gram-negative: *Escherichia coli *and *Pseudomonas putide*; (b) Gram-positive: *Bacillus subtilis *and *Streptococcus lactis*; (c) Fungi: *Aspergillus niger *and *Penicillium *sp*.*; (d) Yeast: *Candida albicans. *

*Media: *Three types of specific media were used in this study: 

*Medium (1): *For bacteria (Nutrient Medium), consisting of (g/L distilled water): peptone, 5 and meat extract, 3. pH was adjusted to 7.0.

*Medium (2): *For fungi (Potato Dextrose Medium), consisting of (g/L distilled water): Infusion from potatoes, 4 and D(+)glucose, 20. pH was adjusted to 5.5.

*Medium (3): *For yeast (Universal Medium), consisting of (g/L distilled water): yeast extract, 3; malt extract, 3; peptone, 5 and glucose, 10. pH was adjusted to 5.5.

For solid media, 2% agar was added. All media were sterilized at 121 °C for 20 min.

Procedure (Filter Paper Diffusion Method) [29]

Proper concentrations of microbial suspensions were prepared from 1 (for bacteria to 3 (for yeast and fungi)-day-old liquid stock cultures incubated on a rotary shaker (100 rpm). In the case of fungi, 5 sterile glass beads were added to each culture flask. The mycelia were then subdivided by mechanical stirring at speed No. 1 for 30 min. Turbidity of microorganisms was adjusted with a spectrophotometer at 350 nm to give an optical density of 1.0 Appropriate agar plates were aseptically surface inoculated uniformLy by a standard volume (ca. 1 mL) of the microbial broth culture of the tested microorganism, namely *E. coli*, *P. putide*, *B. subtilis*, *S. Lactis*, *A. Niger*, *Penicillium sp. *and *C. albicans.*

Whatman No. 3 filter paper discs of 10 mm diameter were sterilized by autoclaving for 15 min at 121 °C. Test compounds were dissolved in 80% ethyl alcohol to give final concentration of 5 μg/mL. The sterile discs were impregnated with the test compounds (5 μg/disc). After the impregnated discs have been air dried, they were placed on the agar surface previously seeded with the organism to be tested. Discs were gently pressed with forceps to insure thorough contact with the media. Three discs were arranged per dish, suitably spaced apart, *i.e*., the discs should be separated by a distance that is equal to or slightly greater than the sum of the diameters of inhibition produced by each disc alone. Each test compound was conducted in triplicate. Plates were kept in the refrigerator at 5 °C for 1 h to permit good diffusion before transferring them to an incubator at 37 °C for 24 h for bacteria and at 30 °C for 72 h for yeast and fungi.

## 4. Conclusions

New thiazolopyrimidines have been synthesized using both conventional methods and microwave assisted conditions. The latter methods proved very efficient in reducing reaction times as well as increasing the overall yield of the reactions. The newly synthesized compounds were tested for their antioxidant and antimicrobial activities. Some compounds showed good or moderate antioxidant activity, whereas other compounds showed weak antimicrobial activity. 
